# Correlation between retinal structure and brain multimodal magnetic resonance imaging in patients with Alzheimer’s disease

**DOI:** 10.3389/fnagi.2023.1088829

**Published:** 2023-02-22

**Authors:** Xiaoli Hao, Weiwei Zhang, Bin Jiao, Qijie Yang, Xinyue Zhang, Ruiting Chen, Xin Wang, Xuewen Xiao, Yuan Zhu, Weihua Liao, Dongcui Wang, Lu Shen

**Affiliations:** ^1^Department of Neurology, Xiangya Hospital of Central South University, Changsha, China; ^2^Department of Radiology, Xiangya Hospital of Central South University, Changsha, China; ^3^National Clinical Research Center for Geriatric Disorders, Central South University, Changsha, China; ^4^Engineering Research Center of Hunan Province in Cognitive Impairment Disorders, Central South University, Changsha, China; ^5^Hunan International Scientific and Technological Cooperation Base of Neurodegenerative and Neurogenetic Diseases, Changsha, China; ^6^Key Laboratory of Hunan Province in Neurodegenerative Disorders, Central South University, Changsha, China; ^7^Key Laboratory of Organ Injury, Aging and Regenerative Medicine of Hunan Province, Changsha, China

**Keywords:** Alzheimer’s disease, retina, visual pathway, multimodal magnetic resonance imaging, biomarker

## Abstract

**Background:**

The retina imaging and brain magnetic resonance imaging (MRI) can both reflect early changes in Alzheimer’s disease (AD) and may serve as potential biomarker for early diagnosis, but their correlation and the internal mechanism of retinal structural changes remain unclear. This study aimed to explore the possible correlation between retinal structure and visual pathway, brain structure, intrinsic activity changes in AD patients, as well as to build a classification model to identify AD patients.

**Methods:**

In the study, 49 AD patients and 48 healthy controls (HCs) were enrolled. Retinal images were obtained by optical coherence tomography (OCT). Multimodal MRI sequences of all subjects were collected. Spearman correlation analysis and multiple linear regression models were used to assess the correlation between OCT parameters and multimodal MRI findings. The diagnostic value of combination of retinal imaging and brain multimodal MRI was assessed by performing a receiver operating characteristic (ROC) curve.

**Results:**

Compared with HCs, retinal thickness and multimodal MRI findings of AD patients were significantly altered (*p* < 0.05). Significant correlations were presented between the fractional anisotropy (FA) value of optic tract and mean retinal thickness, macular volume, macular ganglion cell layer (GCL) thickness, inner plexiform layer (IPL) thickness in AD patients (*p* < 0.01). The fractional amplitude of low frequency fluctuations (fALFF) value of primary visual cortex (V1) was correlated with temporal quadrant peripapillary retinal nerve fiber layer (pRNFL) thickness (*p* < 0.05). The model combining thickness of GCL and temporal quadrant pRNFL, volume of hippocampus and lateral geniculate nucleus, and age showed the best performance to identify AD patients [area under the curve (AUC) = 0.936, sensitivity = 89.1%, specificity = 87.0%].

**Conclusion:**

Our study demonstrated that retinal structure change was related to the loss of integrity of white matter fiber tracts in the visual pathway and the decreased LGN volume and functional metabolism of V1 in AD patients. Trans-synaptic axonal retrograde lesions may be the underlying mechanism. Combining retinal imaging and multimodal MRI may provide new insight into the mechanism of retinal structural changes in AD and may serve as new target for early auxiliary diagnosis of AD.

## Introduction

Alzheimer’s disease (AD) is the most common type of senile dementia and is characterized by a progressive deterioration of cognitive function over time (Ge et al., 2021). The pathology of AD is featured by extracellular amyloid plaques formed by the aggregation of amyloid-β (Aβ) and neuronal fibrillary tangles (NFT) formed by abnormally phosphorylated tau. Studies have demonstrated that the pathological changes of AD precede the onset of clinical symptoms 15–20 years (Drew, 2018). Therefore, early diagnosis of AD is particularly important in order to achieve early intervention and treatment. Currently, Aβ and tau proteins are mainly quantified by positron emission tomography (PET) or cerebrospinal fluid (CSF) (Arvanitakis et al., 2019; Jiao et al., 2021). However, these tests are invasive and expensive, pending the discovery of new universally applicable early diagnostic markers.

The retina shares similar neurobiology of neuronal cells and microvasculature with those in central nervous system (CNS), thus, changes in retina structure and function may serve as a window to access the changes in CNS (Liao et al., 2021; Zhang et al., 2022). In recent years, a number of clinical studies have shown that AD patients have functional visual defects and anatomical changes in retinal structure. In fact, about 58% patients develop visual problems before the initial symptoms of AD, such as impaired visual acuity, color perception, contrast sensitivity, and visual field (Mendez et al., 1990; Mendola et al., 1995; Chiquita et al., 2019). Optical coherence tomography (OCT) is a non-invasive technique for cross-sectional imaging of the internal structure of the retina, primarily to visualize morphological changes in the retina and to show retinal layering. Studies using OCT technology have demonstrated that the thickness of the peripapillary retinal nerve fiber layer (pRNFL) and macular ganglion cell layer (GCL) in patients with early AD and mild cognitive impairment (MCI) are significantly reduced (Criscuolo et al., 2018). Recent studies found that retinal thickness correlated with concentrations of AD biomarkers in CSF and PET-Aβ uptake values (Golzan et al., 2017; Santangelo et al., 2020). Moreover, after taking curcumin, deposition of Aβ protein can be detected in the retina of patients with AD by non-invasive fluorescence imaging techniques (Koronyo et al., 2017). Accumulation of tau protein in the retina of AD mice was observed before the onset of symptoms (Chiasseu et al., 2017). Our team’s previous study also found that the retinal thickness reduced significantly in AD patients, which was correlated with the concentration of Aβ and tau in CSF (Wang X. et al., 2022). These above studies indicate that changes in retinal structure may serve as an early diagnostic biomarker for AD. However, the retinal structural changes are not specific to AD (Cheung et al., 2021; Snyder et al., 2021), their diagnostic and prognostic values in AD are still under debate.

Multimodal magnetic resonance imaging (MRI), dividing as structural imaging and functional imaging, reflect the structure and intrinsic activity of the brain (Zhou et al., 2021). Previous studies found that volumes of hippocampus were reduced in AD (Mu and Gage, 2011). Hippocampal atrophy can also distinguish AD patients from healthy controls and those who will convert from MCI to AD from non-converters (Teipel et al., 2015; Wang H. et al., 2022). In addition, recent study observed the degeneration of lateral geniculate nucleus (LGN) in AD patients (Erskine et al., 2016). Hippocampal atrophy and LGN degeneration may act as early changes in the progenesis of AD, but their relationship with retinal structure remains vague. Diffusion tensor imaging (DTI) is based on water diffusion within the brain that can detect early white matter microstructure changes. After software post-processing, DTI can visualize the visual pathways. The diffusion metrics of DTI include fractional anisotropy (FA), mean diffusivity (MD), radial Diffusivity (RD), and axial diffusivity (DA) (Bosch et al., 2012; Zhang et al., 2014). The loss of nerve fibers and neurotrophic tissue atrophy lead to the decrease of FA and the increase of MD, which are generally considered as indicators of axonal damage (Altıntaş et al., 2017). A study on the relationship between the integrity of whole brain white matter and the thickness of retinal layers in early AD patients found that the FA value of whole brain white matter fiber tracts was positively correlated with the thickness of inner nuclear layer (INL) (Alves et al., 2019). Nevertheless, the study did not separate the FA values of the visual pathways independently, and no association between FA values and thickness of GCL or pRNFL was observed.

Despite functional imaging has not yet been established for routine clinical use, studies confirm that most AD patients have irreversible pathological changes in the brain prior to the appearance of structural abnormalities, and functional changes may precede structural abnormalities (Wang et al., 2013). In recent years, blood oxygen level-dependent (BOLD) functional MRI (fMRI) has been introduced into the field of early AD research (Wierenga and Bondi, 2007). Resting-state fMRI (rs-fMRI) is a new functional imaging technique that indirectly reflects neuronal activity by measuring BOLD signals. In which, fractional amplitude of low frequency fluctuations (fALFF) can reflect spontaneous neuronal activity in brain regions and physiological state of the brain (Xi et al., 2012). Weiler et al. (2014) found that ALFF was reduced in the temporal region of MCI patients and in the posterior cingulate gyrus of mild AD patients. These data manifest functional deficits of certain brain regions in early AD patients. At present, there is still a lack of research on the correlation between retinal structure and brain activity changes in AD patients.

Previous studies have attributed visual deficits in AD patients to degenerative damage in the primary visual cortex (V1) (Mendez et al., 1990; Leuba and Kraftsik, 1994; Armstrong, 1996; Jorge et al., 2020), but several recent studies have shown that visual cortex is relatively unaffected by Aβ and Tau protein pathology (Frisoni et al., 2010; Coppola et al., 2015). This indicates that visual deficits cannot simply be explained by visual cortex dysfunction, implying that other mechanisms may exist that contribute to visual problems in AD patients.

Although retinal structural changes and brain MRI have been explored as possible biomarkers for the early diagnosis of AD, their correlation and the internal mechanism of retinal structural changes remain elusive. Besides, the retinal structural changes are not specific to AD. To fill these gaps in knowledge, we combined OCT and multimodal MRI techniques to explore the possible correlation between retinal structure and visual pathway, brain structure, intrinsic activity changes in AD patients, as well as to evaluate their ability to auxiliary diagnose AD.

## Materials and methods

### Participants

The study included 49 AD patients and 48 healthy controls (HCs) matched by age, gender from the Department of Neurology at Xiangya Hospital of Central South University, between March 2020 and May 2022. The subjects were between 50 and 80 years of age, and the AD patients met the diagnostic criteria of “probable AD” in the 2011 edition of the National Institute on Aging and Alzheimer’s Association (NIA-AA) guidelines (McKhann et al., 2011). The exclusion criteria of the study were as follows: (1) Best-corrected visual acuity (BCVA) >6.00 D or with astigmatism >3.00 D, and (3) intraocular pressure (IOP) >21 mmHg. All participants were free of other neurologic, psychiatric disorders, and systemic diseases that may affect retina (such as diabetes mellitus and uncontrolled hypertension), as well as ocular diseases (including cataract, glaucoma, uveitis, epiretinal membrane, age-related macular degeneration, macular hole, eye trauma, and any eye surgery). The Ethics Committee of Xiangya Hospital of Central South University approved this study, and all subjects signed written informed consent.

### General data collection

General information was collected on all subjects, including name, age, gender, years of education, the history of ocular disease and underlying medical conditions. All subjects were required to undergo cognitive assessments, including the mini-mental state examination (MMSE), Montreal cognitive assessment scale (MOCA), and clinical dementia rating scale (CDR). Besides, intraocular pressure (IOP) and best-corrected visual acuity (BCVA) examinations were necessary.

### Optical coherence tomography scanning

We performed two operations on both eyes of each subject using the Heidelberg Spectralis-OCT instrument (Heidelberg Engineering, Germany).

(1) Intensive horizontal scanning of the central macular area of the retina: 31 B-scans (each B-scan consists of 768 A-scans) were performed within the 6-mm diameter grid for the Early Treatment of Diabetic Retinopathy Study (ETDRS), and the macula thickness was measured in the ETDRS map [average of four quadrants of fovea (Ø 1 mm), inner ring (Ø 3 mm), and outer ring (Ø 6 mm)]. In the macular region, the inner and outer ring layers were segmented and analyzed with Heidelberg segmentation software, and the average macular thickness and the thickness of the following retinal layers were calculated: Macular RNFL (mRNFL), GCL, inner plexiform layer (IPL), INL, outer plexiform layer (OPL), outer nuclear layer (ONL), and retinal pigment epithelium (RPE).
(2) Circumferential scanning of the pRNFL: The pRNFL thickness was measured by the scanning technician in a circular scan over a 3.4 mm diameter area centered on the optic disc. For each pRNFL, 768 A-scans were performed with an ART value (average number of scans) of 100. pRNFL thickness was measured in each sector provided by Heidelberg software, namely global (G), superior quadrant (S), temporal quadrant (T), inferior quadrant (I), and nasal quadrant (N). All scans were performed according to OSCAR-IB consensus criteria (Tewarie et al., 2012) and were manually corrected by the scanning technician (ZXY) in case of unknown diagnosis to find obvious segmentation errors.

### MR imaging acquisition

All MRI experiments were carried out on a 3T Siemens Prisma MRI scanner (Prisma, Siemens, Germany) with a 64-channel head/neck coil. Prior to imaging acquisition, participants were instructed to lie still, to let their mind wander, and to keep eyes closed once the scanning started. MRI-safe soft ear plugs were put into their ears to provide hearing protection. And the gap between their head and the coil was filled with foam paddings to minimize head movements.

The three-dimensional T1-weighted images (3D-T1WI) were acquired using a magnetization prepared rapid acquisition gradient echo (MPRAGE) sequence with the following parameters: Sagittal, 176 slices, slice thickness (ST) = 1 mm, repetition time (TR) = 2,300 ms, echo time (TE) = 2.98 ms, inversion time (TI) = 900 ms, data matrix = 248 × 256, field of view (FOV) = 248 × 256 mm^2^, and flip angle (FA) = 9°.

DTI images were obtained using a single-shot echo-planar imaging (EPI) sequence, whose parameters were configured as 70 continuous axial-oblique (parallel to the anterior-posterior commissural line) slices, 64 acquisitions with diffusion weighting (*b* = 1,000 s/mm2, 1 acquisition for each of 64 non-collinear diffusion sensitization gradients) together with three acquisitions without diffusion weighting (*b* = 0 s/mm2), *ST* = 2 mm, *TR* = 4,200 ms, *TE* = 68 ms, data matrix = 132 × 132, and *FOV* = 264 mm × 264 mm.

Resting-state fMRI data were collected using a gradient echo EPI sequence under an eye-closed condition. The online configuration of scanning parameters was specified as 75 continuous axial-oblique (parallel to the anterior-posterior commissural line) slices, *ST* = 2 mm, *TR* = 2,000 ms, *TE* = 30 ms, data matrix = 94 × 94, *FOV* = 220 mm × 220 mm, *FA* = 60°, and 240 volumes in total.

### Region of interest segmentation

The segmentations of our regions of interest (ROIs) were carried out on 3D T1 images using automated programs in FreeSurfer (v7.0).^1^ First, recon-all, the main FreeSurfer stream, was executed. It completed mainly the following processing stages: (1) Motion correction, (2) intensity normalization, (3) Talairach transformation, (4) non-brain tissue removal, (5) volumetric registration and labeling, (6) white matter segmentation, (7) spherical mapping and registration, and (8) cortical parcellation. Next, ROIs of hippocampus, LGN, and V1 were reconstructed by running the bash scripts of segmentHA_T1.sh (Iglesias et al., 2015), segmentThalamicNuclei.sh (Iglesias et al., 2018), and the recon-all -label-v1 (Hinds et al., 2008), respectively.

### Probabilistic tractography

As suggested by Puzniak et al. (2021), to perform tractography of the visual system, the individual structures of optic chiasm, LGN, and V1 need to be provided at least. In our case, the locations of these structures were estimated automatedly as outlined above. To be specific, optic chiasm, LGN, and V1 were defined as the region with a label identifier of 85 from the aseg file, regions with label identifiers of 8,109 (L) and 8,209 (R) from the segmentation file of the thalamic nuclei, and the region with a label identifier of one from the threshold label file of V1, respectively. All these regions of interest were then transformed from fsaverage to native diffusion space through a two-step registration procedure. First, the segmentation files were registered to individual T1-weighted images through tkregister2. Next, the T1-weighted images were registered to individual FA images using a linear warping algorithm (Supplementary Figure 1).

All diffusion image preprocessing and fiber tracking were conducted using FMRIB Software Library (FSL v6.0).^2^ The preprocessing steps were consisted of correction of motion and eddy current distortion, brain extraction, and local fitting of diffusion tensors. Probabilistic tractography was performed by employing the FDT toolbox. To calculate the probability of fiber connections from optic chiasm to LGN (the optic tract) and from LGN to V1 (the optic radiations), the algorithm was run using default parameters: curvature threshold = 0.2 mm, maximum number of steps per sample = 2,000, length of each step = 0.5 mm, and number of sample tracts = 5,000. To limit cross-hemispheric fiber bundles, tractography was performed separately for the left and right hemisphere by applying the contralateral target as an exclusion mask. The mean diffusion values (FA, MD, DA, and RD) of the optic tract and optic radiation fiber tracts were then extracted.

### Fractional amplitude of low frequency fluctuations analysis

Fractional amplitude of low frequency fluctuations was calculated using the Data Processing and Analysis for Brain Imaging (DPABI V6.0) (Yan et al., 2016), a MATLAB toolbox developed based on Statistical Parametric Mapping (SPM12).^3^ The resting-state fMRI images were first preprocessed through the following steps: (1) Format conversion from DICOM to NIFTI, (2) removal of the first 10 time points, (3) slice timing, (4) realignment, (5) co-registration to the skull stripped T1-weighted image, (6) nuisance covariates (Friston 24 head motion regressors, as well as white matter, ventricular CSF and global signals) regression, and (6) spatial normalization to the MNI 152 template. The preprocessed voxel-wise time series were then transformed to frequency domain *via* fast Fourier transform (FFT). fALFF was then calculated as the ratio of power spectrum of a specified frequency band (0.01–0.08 Hz) to that of the entire detectable frequency range (Zou et al., 2008). For standardization purposes, Fisher’s r-to-z transformation was then performed to individual fALFF maps.

The mask file of V1 in individual T1 space was transformed to the MNI space by applying the same transform parameters generated by the aforementioned preprocessing step of spatial normalization. The regional mean of z-score fALFF maps for V1 was then extracted.

### Statistical analysis

The average values of bilateral hemispheric data were calculated, and the Shapiro–Wilk test was used to test the distribution of normality of the data. Group comparisons were performed using the independent sample *t*-test or the Mann–Whitney *U*-test as appropriate. Categorical data were analyzed using the χ2 test.

Spearman correlation analysis was used to assess the correlation between retinal structure and multimodal MRI findings in AD and HC groups, respectively.

Multiple linear regression model was developed based on the results of correlation analysis to explore the effects of multimodal MRI findings on retinal thickness in AD patients, and the results were expressed as standardized coefficients β and their corresponding 95% confidence intervals (CI), adjusting for covariates such as gender, age, years of education and dementia degree.

Diagnostic accuracy was performed using receiver operating characteristic (ROC) curve analysis. The area under the curve (AUC) and representative optimal sensitivity and specificity were used to evaluate the performance of the models. The Delong’s test was conducted to compare the difference of two different diagnostic models.

All statistical tests were performed using the standard statistical software SPSS (version 26.0) and MedCalc (version 20.115), using a two-tailed test, and *p*-values < 0.05 were considered statistically significant. GraphPad Prism (version 9.0) and Origin (version 2022) was used to plot the graphs.

## Results

A total of 97 subjects were included, including 49 AD patients and 48 HCs. Age and gender were matched between AD patients and HCs. No significant differences were observed in IOP and BCVA between two groups. About 33% of AD patients (16 patients) complained with visual deficits, including impaired visual acuity, color perception and visual field, compared with 6% of HCs (3 controls). The AD group had a shorter mean duration of education. There were significant differences in cognitive scores, as expected (Table 1).

**TABLE 1:** The demographic and clinical characteristics of the subjects.

Variables	AD	HCs	*P*-value
Number	49	48	NA
Age, year	64.88 ± 7.24	63.42 ± 5.31	0.261
Sex, m/f	19/30	17/31	0.732
Years of education	9.00 ± 4.22	10.85 ± 3.38	0.018*
MMSE	16.92 ± 7.56	28.00 ± 1.49	<0.001***
MOCA	10.88 ± 6.34	22.04 ± 3.23	<0.001***
CDR	1.15 ± 0.55	0	<0.001***
IOP (mmHg)	15.14 ± 1.77	15.29 ± 1.70	0.674
BCVA	1.19 ± 0.25	1.14 ± 0.26	0.391
Visual deficits (%)	33%	6%	NA

AD, Alzheimer’s disease; HCs, health controls; MMSE, mini-mental state examination; MoCA, montreal cognitive assessment; CDR, clinical dementia rating; IOP, intraocular pressure; BCVA, best-corrected visual acuity. *p < 0.05 and ***p < 0.001.

### Group difference in retinal structure and multimodal MRI

**FIGURE 1:**
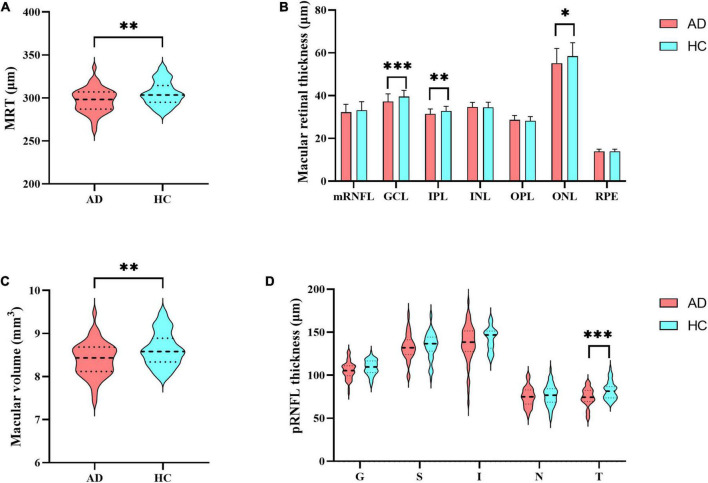
Differences in retinal thickness. **(A)** Mean retinal thickness; **(B)** the thickness of retinal layers in the macular region: mRNFL, GCL, IPL, INL, OPL, ONL and RPE; **(C)** macular volume; **(D)** peripapillary RNFL (pRNFL) thickness: global thickness (G), superior quadrant (S), inferior quadrant (I), nasal quadrant (N), temporal quadrant (T). MRT, mean retinal thickness; mRNFL, macular retinal nerve fiber layer; GCL, ganglion cell layer; IPL, inner plexiform layer; INL, inner nuclear layer; OPL, outer plexiform layer; ONL, outer nuclear layer; RPE, retinal pigment epithelium; pRNFL, peripapillary RNFL; G, global thickness; S, superior quadrant; I, inferior quadrant; N, nasal quadrant; T, temporal quadrant. Significant differences are indicated by square brackets. *Denotes *p* < 0.05, ^**^denotes *p* < 0.01, and ^***^denotes *p* < 0.001.

Compared to HCs, AD patients presented significantly thinner mean retinal thickness, macular volume, GCL thickness, IPL thickness, and ONL thickness, reduced by 2.70%, 2.67%, 5.82%, 4.06%, and 5.61%, respectively (Figure 1 and Supplementary Table 1). Additionally, comparison of pRNFL thickness revealed 9.66% reduction of temporal quadrant pRNFL thickness in AD group (*p* < 0.001), indicating that pRNFL thickness was preferentially reduced in the temporal quadrant (Figure 1).

**FIGURE 2:**
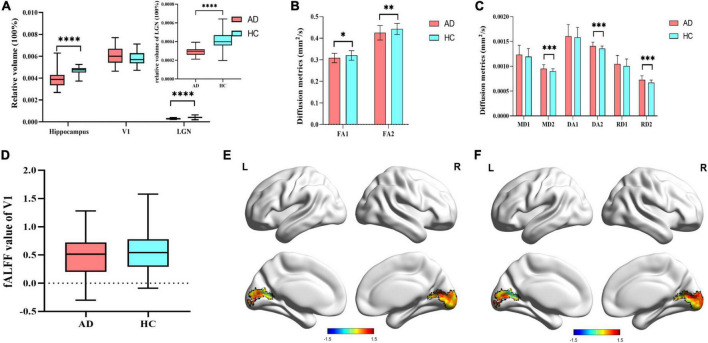
Differences in multimodal MRI. **(A)** Difference of 3D-T1WI results. **(B)** Difference of FA value in the visual pathway. FA1 represent FA value of optic tract, and FA2 represent FA value of optic radiation. **(C)** Difference of other diffusion metrics in the visual pathway. MD1, DA1, and RD1 represent MD, DA, and RD values of optic tract, respectively. MD2, DA2, and RD2 represent MD, DA, and RD values of optic radiation, respectively. **(D)** Difference of fALFF value of V1 in AD group and HCs group; **(E)** fALFF value of V1 in AD patients; **(F)** fALFF value of V1 in HCs. V1, primary visual cortex; LGN, lateral geniculate nucleus; FA, fractional anisotropy; MD, mean diffusivity; DA, axial diffusivity; RD, radial diffusivity; fALFF, fractional amplitude of low frequency fluctuations. Significant differences are indicated by square brackets. *Denotes *p* < 0.05, ^**^denotes *p* < 0.01, ^****^means *p* < 0.0001.

Compared to HCs, there was a significant reduction of the relative volume of hippocampus (16.88%, *p* < 0.001) and LGN (29.27%, *p* < 0.001) in AD patients. No statistical change was found in the volume of V1 between the two group. In terms of the diffusion metrics of the visual pathway, we found significant decreases in the FA value of optic tract (3.75%, *p* = 0.011) and optic radiation (4.06%, *p* = 0.006) in AD patients. Measures of MD, DA and RD values increased among optic tract and optic radiation, but only met statistical significance in the optic radiation (Figure 2 and Supplementary Table 2). Besides, there were no significant differences in the fALFF value of V1 between AD patients and HCs (Figure 2).

### Correlation between retinal structure and multimodal MRI

**FIGURE 3:**
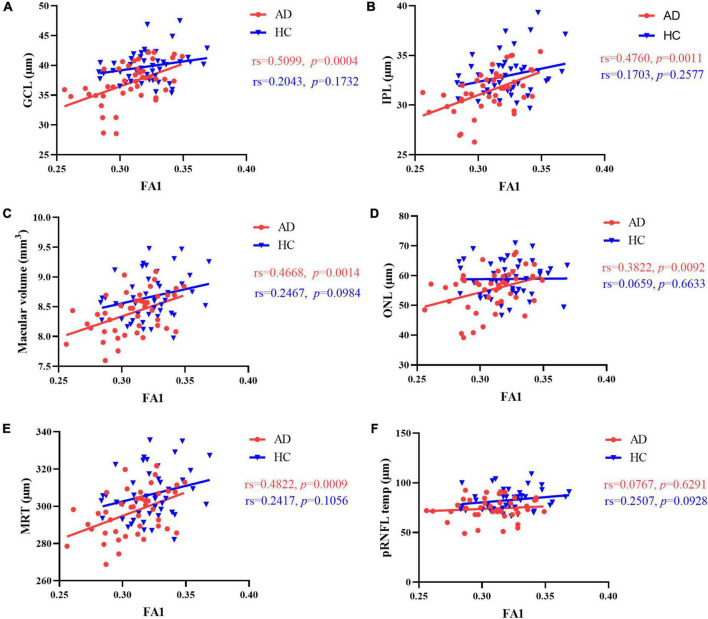
Correlation between FA value of optic tract and retinal thickness. In AD patients, there was a significant positive correlation between FA value of optic tract and **(A)** GCL thickness, **(B)** IPL thickness, **(C)** macular volume, **(D)** ONL thickness and **(E)** mean retinal thickness. **(F)** No correlation was found between FA value of optic tract and temporal quadrant pRNFL thickness. There was no correlation between FA value of optic tract and retinal thickness in HCs. MRT, mean retinal thickness; GCL, ganglion cell layer; IPL, inner plexiform layer; ONL, outer nuclear layer; pRNFL, peripapillary retinal nerve fiber layer; FA1, fractional anisotropy of the optic tract.

**FIGURE 4:**
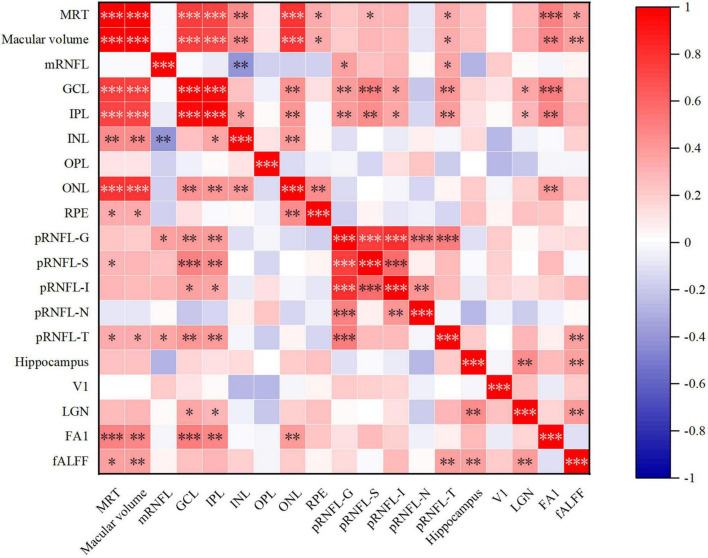
Heat map of correlation between retinal structure and multimodal MRI in AD group. MRT, mean retinal thickness; mRNFL, macular retinal nerve fiber layer; GCL, ganglion cell layer; IPL, inner plexiform layer; INL, inner nuclear layer; OPL, outer plexiform layer; ONL, outer nuclear layer; RPE, retinal pigment epithelium; pRNFL, peripapillary RNFL; G, global thickness; S, superior quadrant; I, inferior quadrant; N, nasal quadrant; T, temporal quadrant; V1, primary visual cortex; LGN, lateral geniculate nucleus; FA1, fractional anisotropy value of optic tract; fALFF, fractional amplitude of low frequency fluctuations. *p < 0.05, ^**^p < 0.01, and ^***^p < 0.001.

Results of Spearman’s correlation test revealed that the FA value of optic tract was highly correlated with mean retinal thickness, macular volume, GCL thickness, IPL thickness, ONL thickness, but not temporal quadrant pRNFL thickness (Figures 3,4 and Supplementary Table 3). There was no significant correlation between the FA value of optic tract and the thickness of any retinal layers in HCs (Supplementary Table 4).

**FIGURE 5:**
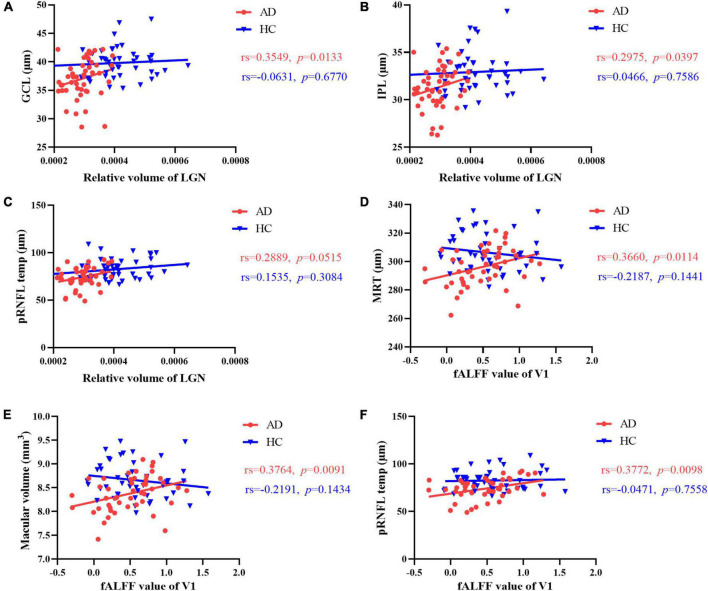
Correlation between brain structure, intrinsic activity changes and retinal thickness. The relative volume of LGN was correlated with **(A)** GCL thickness and **(B)** IPL thickness in AD patients. **(C)** No significant correlation was observed between temporal quadrant pRNFL thickness and the volume of LGN in the two group. In AD patients, there was a significant positive correlation between the fALFF value of V1 and **(D)** mean retinal thickness, **(E)** macular volume, **(F)** temporal quadrant pRNFL thickness. No correlation was found between fALFF value of V1 and the retinal thickness of any layer in HCs. GCL, ganglion cell layer; IPL, inner plexiform layer; MRT, mean retinal thickness; pRNFL, peripapillary retinal nerve fiber layer; LGN, lateral geniculate nucleus; fALFF, fractional amplitude of low frequency fluctuations; V1, primary visual cortex.

In AD group, the relative volume of LGN was correlated with GCL thickness and IPL thickness in AD patients (Figures 4, 5 and Supplementary Table 3). No significant correlation was observed between the retinal thickness of any layer and the volume of hippocampus or V1 in the two group (Supplementary Table 4).

Furthermore, we found a significant positive correlation between the fALFF value of V1 and mean retinal thickness, macular volume, temporal quadrant pRNFL thickness in AD group (Figures 4, 5 and Supplementary Table 3). In HCs, no correlation was found between the fALFF value of V1 and the retinal thickness of any layer (Supplementary Table 4).

Based on the correlation results, we carried out multiple linear regression model to explore the effects of multimodal MRI findings on retinal structure in AD patients. After adjusted for gender, age, years of education and dementia degree, the FA value of optic tract demonstrated significant correlation with mean retinal thickness, macular volume, GCL thickness, IPL thickness, ONL thickness in AD patients (Table 2). In addition, the fALFF value of V1 were correlated with temporal quadrant pRNFL thickness (β = 0.345, *p* = 0.044) (Table 2).

**TABLE 2:** Multiple linear regression model of the effect of multimodal imaging on retinal structure in AD patients.

Variable	β	B	95% CI	*P*-value
**GCL thickness (μm)**				
FA1	0.474	71.708	(30.525, 112.890)	0.001**
Gender	-0.115	-0.771	(−2.785, 1.243)	0.443
Age	-0.117	-0.052	(−0.173, 0.068)	0.385
Years of education	-0.302	-0.250	(−0.496, -0.004)	0.047*
Dementia degree	-0.082	-0.473	(−2.034, 1.088)	0.543
**Adjusted. R^2^ = 0.255**				
**IPL thickness (μm)**				
FA1	0.489	46.578	(20.875, 72.280)	0.001**
Gender	-0.232	-0.980	(−2.237, 0.277)	0.123
Age	-0.164	-0.046	(−0.121, 0.029)	0.225
Years of education	-0.299	-0.155	(−0.309, -0.002)	0.047*
Dementia degree	0.004	0.016	(−0.958, 0.991)	0.973
**Adjusted. R^2^ = 0.266**				
**Macular volume (mm^3^)**				
FA1	0.471	7.466	(3.266, 11.665)	0.001**
Gender	-0.278	-0.197	(−0.399, 0.004)	0.055
Age	-0.074	-0.003	(−0.017, 0.010)	0.604
Years of education	-0.254	-0.022	(−0.048, 0.003)	0.085
Dementia degree	-0.210	-0.130	(−0.291, 0.031)	0.110
fALFF	0.263	0.252	(−0.028, 0.533)	0.076
**Adjusted. R^2^ = 0.344**				
**pRNFL temp (μm)**				
fALFF	0.345	10.197	(0.266, 20.128)	0.044*
Gender	0.159	3.510	(−3.485, 10.504)	0.317
Age	-0.028	-0.041	(−0.529, 0.447)	0.867
Years of education	-‘0.031	-0.082	(−0.959, 0.794)	0.850
Dementia degree	0.010	0.187	(−5.593, 5.966)	0.948
**Adjusted. R^2^ = 0.063**				
**ONL thickness (μm)**				
FA1	0.317	98.613	(14.514, 182.712)	0.023*
Gender	-0.296	-4.088	(−8.200, 0.024)	0.051
Age	-0.230	-0.211	(−0.458, 0.035)	0.091
Years of education	-0.260	-0.441	(−0.944, 0.061)	0.083
Dementia degree	-0.337	-3.993	(−7.181, -0.805)	0.015*
**Adjusted. R^2^ = 0.265**				
**MRT (μm)**				
FA1	0.471	264.966	(116.171, 413.761)	0.001**
Gender	-0.281	-7.073	(−14.213, 0.068)	0.052
Age	-0.070	-0.117	(−0.591, 0.357)	0.620
Years of education	-0.255	-0.792	(−1.697, 0.113)	0.084
Dementia degree	-0.209	-4.593	(−10.291, 1.105)	0.111
fALFF	0.262	8.929	(−1.010, 18.867)	0.077
**Adjusted. R^2^ = 0.344**				

GCL, ganglion cell layer; IPL, inner plexiform layer; pRNFL, peripapillary retinal nerve fiber layer; ONL, outer nuclear layer; MRT, mean retinal thickness; FA1, fractional anisotropy (FA) value of the optic tract; fALFF, fractional amplitude of low frequency fluctuations. *p < 0.05 and **p < 0.01.

### Performance of combined diagnostic models

**FIGURE 6:**
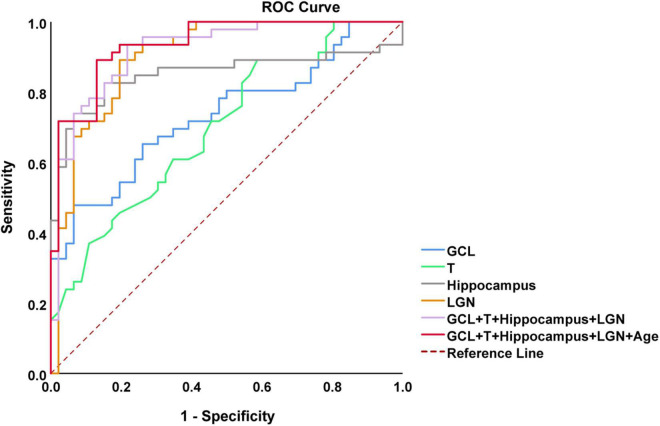
Receiving operating characteristic (ROC) curves analysis of combined OCT and multimodal MRI for diagnosis of AD. The model combining thickness of GCL and temporal quadrant pRNFL (T), volume of hippocampus and lateral geniculate nucleus (LGN), and age showed the best performance to identify AD patients [area under the curve (AUC) = 0.936, sensitivity = 89.1%, specificity = 87.0%]. AUC, area under the curve; ROC, receiver operating characteristic curve; OCT, optical coherence tomography; GCL, ganglion cell layer; pRNFL, peripapillary retinal nerve fiber layer.

In the analysis of differences in retinal structure and multimodal MRI, we found the four parameters (thickness of GCL and temporal quadrant pRNFL, and volume of hippocampus and LGN) were significantly reduced in AD patients (*p* < 0.001). Based on the results, we carried out further analyses to evaluate the performance of the combination of these four parameters to differentiate AD patients from HCs by plotting ROC curves and calculating AUC. After adjusted by Age, the combined model showed the best performance to discriminate between AD patients and HCs (AUC = 0.936, sensitivity = 89.1%, specificity = 87.0%) (Figure 6).

## Discussion

Our study evaluated the changes of retinal thickness and multimodal MRI findings in AD patients. We also correlated OCT findings with multimodal MRI imaging in the study, and the results showed that retinal structure change was related to the loss of integrity of white matter fiber tracts in the visual pathway and the decreased LGN volume and functional metabolism of V1 in AD patients. In addition, a combined model with high auxiliary diagnostic efficacy of AD was generated, including thickness of GCL and temporal quadrant pRNFL, volume of hippocampus and LGN, and age, which can be applied to assist in the identification of probable AD patients. To the best of our knowledge, this is the first study on the correlation of retinal structure and brain multimodal MRI in AD patients.

Here we have shown, in agreement with previous studies, that the mean retinal thickness, macular volume, GCL, IPL, ONL, and temporal quadrant pRNFL thickness were significantly reduced in AD patients. Several previous studies have shown that pRNFL and GC-IPL layers are significantly thinner in early AD patients, and the latest studies indicated that macular GC-IPL layer may be more sensitive than pRNFL thickness (Cheung et al., 2021). Asanad et al. (2019) conducted the first fundus histology study on patients with AD using postmortem human tissues, and the results showed a gradient of retinal thickness reduction, temporal quadrant pRNFL, GCL, and IPL layers were thinned the most, followed by INL and ONL layers. In addition, AD mouse model experiment indicated that the degeneration of dendrites of retinal ganglion cells confined to the plexus layer in the retina preceded their cell loss (Williams et al., 2013). These findings, together with the findings of our study, suggest that changes in retinal structure may serve as an early diagnostic biomarker for AD.

Our results showed that the LGN volume of AD patients were significantly reduced. The changes of LGN in AD patients were rarely studied. Previous studies have found pathological changes such as significantly increased Aβ protein, decreased number of small cell neurons, and large-cell gliosis in LGN of AD patients, whereas the significance of LGN degeneration in AD patients has not been clarified (Erskine et al., 2016). Besides, we observed that volume and neuronal activity of V1 was not changed. Evidence from postmortem autopsy studies suggested that the visual cortex was not severely affected in patients with AD (Leuba and Saini, 1995). These studies confirmed that visual cortex was not affected in AD patients. Regarding to the diffusion metrics of the visual pathway, the first study by Nishioka et al. (2015) showed the changes of white matter fiber tracts in the visual pathway of AD patients. Reduction in FA and increases in MD DA and RD were observed in the optic nerve, less extensive similar changes were also found in the optic tract of AD patients. Likewise, our study showed the same changes in the optic tract. Besides, since the optic radiation is the axon of the geniculate striatum (Ogawa et al., 2014), and LGN volume of AD patients were significantly reduced. More significantly increases in MD, AD and RD and reduction in FA were found within optic radiation. These diffusional differences mirror white matter damage of visual pathway. The above results indicate that white matter damage extends to the visual system, which may contribute to the visual deficits in AD patients.

Studies have shown that thinning of GC-IPL in AD patients is associated with volume reduction of occipital and temporal lobes (Ong et al., 2015; Carazo-Barrios et al., 2021). In contrast with previous work, no correlation was observed between retinal structure and the volume of hippocampus or V1 in AD patients in our study, probably because most of the patients included in this study were mild-to-moderate AD patients, and the sample size was relatively small. Interestingly, significant correlation was presented between GC-IPL and the LGN volume, implying the important role of LGN in the retinal structural changes of AD patients. We found that fALFF value of V1 were positively correlated with temporal quadrant pRNFL thickness, indicating that temporal quadrant pRNFL thickness could reflect neuronal activity in V1. Since the foveal fibers occupy most of the temporal quadrant part of the optic nerve papilla (Jansonius et al., 2009), temporal quadrant pRNFL thickness is more sensitive to reflect brain metabolism than other quadrant pRNFL thickness. These findings provide new functional features for the involvement of visual cortex in the development of retinal structural changes.

Remarkably, significant correlations were presented between the FA value of optic tract and mean retinal thickness, macular volume, GCL thickness and IPL thickness in AD patients. As the optic tract is a part of the RGC axon, the change of optic tract may be most related to the loss of RGC, and the RGC cell bodies are mainly in the macular GC-IPL layer (Song et al., 2018), thus explaining the correlation between the FA value of optic tract and the thickness of GC-IPL. However, no correlation was observed between the FA values of visual pathway and the thickness of pRNFL. The cell bodies and dendrites of RGCs are mainly located in the GC- IPL layer of the macula, while axons are clustered in the pRNFL layer. Since the macula contains more than 50% of the total RGCs and the cell bodies of RGCs are 10–20 times larger than the axon diameter, the macular GC-IPL is more sensitive to AD pathology than the pRNFL (Chan et al., 2019). In short, our study demonstrated that structural changes in the retina of AD patients was associated with damage to the white matter fiber tracts of the visual pathway.

In the present study, we found that FA value of optic tract correlated highly with macular volume, GCL and IPL thickness, besides LGN volume was correlated with GCL and IPL thickness in AD patients. Previous studies have confirmed that pathological features of AD can be found in the LGN and superior colliculus (Leuba and Saini, 1995). Thus, we speculated that there might be a trans-synaptic axonal retrograde lesions process in the visual pathway in AD patients. This hypothesis has been confirmed by several previous studies in which Van Buren et al. noted trans-synaptic retrograde lesions in the macaque visual system, and severe loss of ganglion cells in the retina corresponding to the lesion was observed after either surgical injury to the optic chiasma or ablation of the occipital lobe (Vanburen, 1963). Jindahra et al. (2009) demonstrated that both congenital and acquired visual pathway injuries in humans could cause thinning of the retinal nerve fiber layer through the study of patients with posterior knee lesions, which is likely due to the trans-synaptic axonal retrograde degenerative lesions following damage to the visual pathway. Furthermore, den Haan et al. (2018) found a correlation between RNFL thickness and posterior cortical atrophy in AD patients, which covered the cortex involved in visual processing. Consequently, the above studies provide evidence for trans-synaptic axonal retrograde lesions of the visual pathway in AD patients.

Some histopathological studies have identified Aβ protein deposition in the retina in AD animal models as well as in patients with early AD (Gupta et al., 2021). Although the mechanism of Aβ propagation pathology in AD remains unclear, a growing number of findings confirm that Aβ and Tau can be transmitted from one neuron to another along anatomically connected synapses (Lv et al., 2017). Several zoological studies have proved this theory. Nishioka et al. (2019) used transgenic tau mice to observe the diffusion change pattern caused by Aβ injection into LGN, and the change first appeared in the LGN and optic tract, and then in the optic nerve, implying that there is a process of trans-synaptic axonal retrograde lesions in the visual pathway of AD mice. Similarly, Sun et al. (2014) injected Aβ1-42 into the left ventricle of the mouse model, and DTI showed reduced FA and significantly increased RD in the optic nerve and optic tract 2 months later. These studies imply that there may be a process of trans-synaptic axonal retrograde lesions of Aβ pathologic protein in AD patients.

Although numerous studies have explored the possibility of retinal structure as an early diagnostic marker for AD, the diagnostic accuracy of retinal structure alone may not be sufficient due to the low specificity of retinal structural changes (Lemmens et al., 2020). In the current study, an AUC of 0.936 (CI 0.889–0.983) was achieved using a combination of OCT and multimodal MRI data, which improved the ability to assist detecting AD patients. Thus, OCT combined with multimodal MRI maybe serve as a potential auxiliary diagnostic tool for AD. Currently, most studies on the combination of OCT and MRI focus on the correlation between them. Some studies have found that retinal thickness is significantly related to brain volume (López-Cuenca et al., 2022; Mathew et al., 2023), but there is no study on the diagnosis of AD by combination of OCT and MRI yet.

The study also has certain limitations. Firstly, the case group was mostly mild-to-moderate AD patients due to the strong cooperation required for each examination, and the sample size was relatively small, so we failed to stratify AD patients according to the severity. Secondly, this study was a cross-sectional study, which is unable to observe the dynamic changes of each index longitudinally and to elucidate the causal relationship between retinal structure and multimodal MRI. Longitudinal study on retina and relevant brain MRI changes of early stages (such as subjective cognitive impairment, preclinical and prodromal AD and MCI) should be further explored. Thirdly, only a small part of patients involved in this study had undergone lumbar puncture or PET examination, that didn’t reach the minimum sample size. We will explore comparing this combined model with CSF biomarkers and PET scans in future research. Last but not least, only AD patients and controls were included in our study, which indeed lacks diagnostic specificity. In the follow-up research, we will evaluate the retinal structural characteristics of different types of dementia and explore disease-specific diagnostic indicators.

## Conclusion

In summary, our study confirmed that the retinal structure and multimodal MRI findings of AD patients were significantly altered. The retinal structure change was related to the loss of integrity of white matter fiber tracts in the visual pathway and the decreased LGN volume and functional metabolism of V1 in AD patients. We speculate that the damage of white matter fiber tracts in the visual pathway may lead to the loss of retinal ganglion cells through trans-synaptic axonal retrograde lesions, which may be the cause of retinal structural changes in AD patients. Furthermore, our study supported that retinal imaging combined with multimodal MRI can contribute to a classification model for the identification of AD patients.

## Data availability statement

The raw data supporting the conclusions of this article will be made available by the authors, without undue reservation.

## Ethics statement

The studies involving human participants were reviewed and approved by the Ethics Committee of Xiangya Hospital of Central South University. The patients/participants provided their written informed consent to participate in this study.

## Author contributions

XH, WZ, DW, and LS performed the study design, acquisition of data, analysis and interpretation of data, and drafting/revising the manuscript. BJ, QY, RC, YZ, XW, XX, and WL analyzed the data and revised the manuscript for intellectual content. XZ performed the OCT images scan. All authors contributed to the article and approved the submitted version.

## Footnotes

1
http://surfer.nmr.mgh.harvard.edu

2
https://www.fmrib.ox.ac.uk/fsl

3
http://www.fil.ion.ucl.ac.uk/spm
