# Supplementary material for: Correlation between retinal structure and brain multimodal magnetic resonance imaging in patients with Alzheimer’s disease
Source: Front Aging Neurosci. 2023 Feb 22;15:1088829. doi: 10.3389/fnagi.2023.1088829 (PMC9992546; doi:10.3389/fnagi.2023.1088829)
Supplement: Supplementary file 1 [file Data_Sheet_1.docx]

Supplementary Material

## **Supplementary Figures**


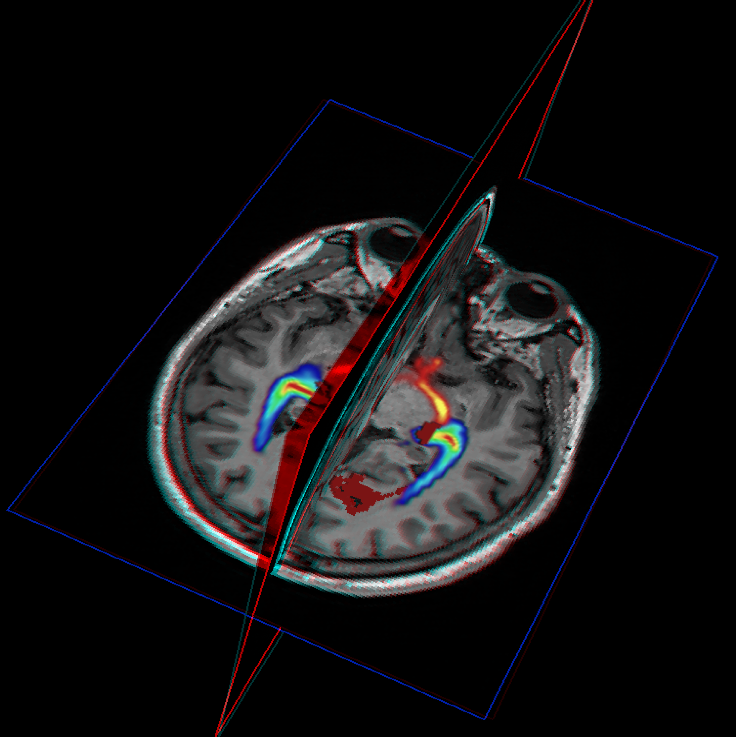


**Supplementary Figure 1.** DTI diagram of visual pathway. Visual pathway reconstruction after probabilistic tractography in subjects. The optic tracts are shown in yellow, the optic radiation is shown in blue, and the ROIs of the optic chiasm, LGN, and V1 are shown in red. DTI: diffusion tensor imaging, ROIs: regions of interest, LGN: lateral geniculate nucleus,V1: primary visual cortex.

**Supplementary Table 1** Differences in retinal thickness between AD patients and HCs

| OCT parameters | AD | HCs | *p* |
| --- | --- | --- | --- |
| Macula, μm |  |  |  |
| MRT | 297.13±14.04 | 305.36±13.93 | 0.005** |
| Macular volume | 8.40±0.40 | 8.63±0.40 | 0.005** |
| mRNFL | 32.19±3.73 | 33.10±4.03 | 0.250 |
| GCL | 37.23±3.59 | 39.53±2.93 | ＜0.001*** |
| IPL | 31.44±2.36 | 32.77±2.25 | 0.006** |
| INL | 34.69±2.14 | 34.52±2.40 | 0.713 |
| OPL | 28.65±2.08 | 28.20±2.00 | 0.285 |
| ONL | 55.15±6.90 | 58.43±6.29 | 0.016* |
| RPE | 13.92 ± 1.03 | 13.92 ± 1.01 | 0.982 |
| pRNFL, μm |  |  |  |
| Global | 105.61±10.22 | 109.21±8.74 | 0.068 |
| Superior | 132.91±15.70 | 134.74±16.11 | 0.578 |
| Inferior | 136.97±23.06 | 142.57±13.32 | 0.150 |
| Nasal | 74.98±11.49 | 76.79±12.51 | 0.464 |
| Temporal | 74.31±10.79 | 82.26±10.47 | ＜0.001*** |

OCT, optical coherence tomography; MRT, mean retinal thickness; mRNFL, macular retinal nerve fiber layer; GCL, ganglion cell layer; IPL, inner plexiform layer; INL, inner nuclear layer; OPL, outer plexiform layer; ONL, outer nuclear layer; RPE, retinal pigment epithelium; pRNFL, peripapillary RNFL. * denotes *p* < 0.05, ** denotes *p* < 0.01, and *** denotes *p* < 0.001.

**Supplementary Table 2** Differences in multimodal MRI findings between AD patients and HCs

| MRI parameters | AD | HCs | *p* |
| --- | --- | --- | --- |
| 3D-T1WI |  |  |  |
| Relative volume of Hippocampus (%) | 0.389±0.073 | 0.468±0.031 | ＜0.001*** |
| Relative volume of LGN (%) | 0.029±0.005 | 0.041±0.009 | ＜0.001*** |
| Relative volume of V1 (%) | 0.608±0.085 | 0.580±0.056 | 0.051 |
| DTI |  |  |  |
| FA1 (mm^2^/s) | 0.308±0.0219 | 0.320±0.022 | 0.011* |
| FA2 (mm^2^/s) | 0.425±0.034 | 0.443±0.025 | 0.006** |
| MD1 (×10^3^ mm^2^/s) | 1.233±0.191 | 1.195±0.162 | 0.302 |
| MD2 (×10^3^ mm^2^/s) | 0.952±0.080 | 0.900±0.048 | ＜0.001*** |
| DA1 (×10^3^ mm^2^/s) | 1.606±0.234 | 1.581±0.201 | 0.588 |
| DA2 (×10^3^ mm^2^/s) | 1.407±0.078 | 1.355±0.052 | ＜0.001*** |
| RD1 (×10^3^ mm^2^/s) | 1.047±0.172 | 1.002±0.145 | 0.176 |
| RD2 (×10^3^ mm^2^/s) | 0.725±0.084 | 0.672±0.051 | ＜0.001*** |
| Rs-fMRI |  |  |  |
| fALFF value of V1 | 0.483±0.362 | 0.568±0.394 | 0.272 |

3D-T1WI, three-dimensional T1-weighted imaging; LGN, lateral geniculate nucleus; V1, primary visual cortex; DTI, diffusion tensor imaging; FA1 represent fractional anisotropy (FA) value of optic tract, and FA2 represent FA value of optic radiation. MD1, DA1 and RD1 represent mean diffusivity (MD), axial diffusivity (DA) and radial diffusivity (RD) values of optic tract respectively. MD2, DA2 and RD2 represent MD, DA and RD values of optic radiation, respectively. Rs-fMRI, Resting-state functional MRI; fALFF, fractional amplitude of low frequency fluctuations.

* denotes *p* < 0.05, ** denotes *p* < 0.01, **** means *p* < 0.0001.

**Supplementary Table 3** Correlation between retinal structure and multimodal MRI in AD group

| Retinal structure | Relative volume of Hippocampus | | Relative volume of V1 | | Relative volume of LGN | | FA value of  optic tract | | fALFF value  of V1 | |
| --- | --- | --- | --- | --- | --- | --- | --- | --- | --- | --- |
|  | r | *p* | r | *p* | r | *p* | r | *p* | r | *p* |
| MRT | 0.258 | 0.076 | 0.009 | 0.951 | 0.277 | 0.057 | 0.482 | <0.001*** | 0.366 | 0.011* |
| Macular volume | 0.256 | 0.079 | 0.016 | 0.912 | 0.283 | 0.051 | 0.467 | 0.001** | 0.376 | 0.009** |
| mRNFL | -0.282 | 0.052 | 0.203 | 0.172 | 0.036 | 0.808 | -0.036 | 0.818 | 0.054 | 0.717 |
| GCL | 0.174 | 0.237 | 0.109 | 0.466 | 0.355 | 0.013* | 0.51 | <0.001*** | 0.249 | 0.091 |
| IPL | 0.139 | 0.347 | 0.04 | 0.792 | 0.298 | 0.039* | 0.476 | 0.001** | 0.284 | 0.053 |
| INL | 0.147 | 0.32 | -0.263 | 0.074 | -0.045 | 0.76 | -0.002 | 0.991 | 0.181 | 0.223 |
| OPL | 0.01 | 0.947 | -0.269 | 0.067 | -0.213 | 0.147 | -0.032 | 0.836 | -0.025 | 0.865 |
| ONL | 0.206 | 0.159 | -0.02 | 0.894 | 0.186 | 0.206 | 0.388 | 0.009** | 0.203 | 0.171 |
| RPE | 0.246 | 0.091 | 0.05 | 0.741 | 0.257 | 0.077 | 0.222 | 0.148 | 0.044 | 0.771 |
| pRNFL-G | -0.109 | 0.47 | 0.201 | 0.185 | 0.027 | 0.858 | 0.128 | 0.419 | 0.159 | 0.291 |
| pRNFL-S | -0.014 | 0.928 | 0.19 | 0.211 | 0.004 | 0.981 | 0.265 | 0.089 | -0.008 | 0.96 |
| pRNFL-I | -0.067 | 0.66 | 0.173 | 0.256 | 0.136 | 0.369 | 0.186 | 0.239 | 0.265 | 0.075 |
| pRNFL-N | -0.263 | 0.078 | -0.046 | 0.764 | -0.196 | 0.193 | -0.044 | 0.782 | 0.031 | 0.836 |
| pRNFL-T | 0.216 | 0.149 | 0.018 | 0.904 | 0.289 | 0.052 | 0.077 | 0.629 | 0.377 | 0.010** |

MRT, mean retinal thickness; mRNFL, macular retinal nerve fiber layer; GCL, ganglion cell layer; IPL, inner plexiform layer; INL, inner nuclear layer; OPL, outer plexiform layer; ONL, outer nuclear layer; RPE, retinal pigment epithelium; pRNFL, peripapillary RNFL; G, global thickness; S, superior quadrant; I, inferior quadrant; N, nasal quadrant; T, temporal quadrant; LGN, lateral geniculate nucleus; V1, primary visual cortex; FA, fractional anisotropy; fALFF, fractional amplitude of low frequency fluctuations. * denotes *p* < 0.05, ** denotes *p* < 0.01, and *** denotes *p* < 0.001.

**Supplementary Table 4** Correlation between retinal structure and multimodal MRI in HCs

| Retinal structure | Relative volume of Hippocampus | | Relative volume of V1 | | Relative volume of LGN | | FA value of  optic tract | | fALFF value  of V1 | |
| --- | --- | --- | --- | --- | --- | --- | --- | --- | --- | --- |
|  | r | *p* | r | *p* | r | *p* | r | *p* | r | *p* |
| MRT | -0.049 | 0.747 | -0.111 | 0.463 | 0.091 | 0.548 | 0.242 | 0.106 | -0.219 | 0.144 |
| Macular volume | -0.054 | 0.719 | -0.103 | 0.494 | 0.09 | 0.551 | 0.247 | 0.098 | -0.219 | 0.143 |
| mRNFL | -0.187 | 0.214 | -0.176 | 0.242 | 0.045 | 0.767 | 0.315 | 0.033* | -0.028 | 0.853 |
| GCL | 0.084 | 0.58 | 0.07 | 0.646 | 0.063 | 0.677 | 0.204 | 0.173 | -0.166 | 0.269 |
| IPL | 0.061 | 0.687 | -0.002 | 0.99 | 0.047 | 0.759 | 0.17 | 0.258 | -0.209 | 0.162 |
| INL | -0.115 | 0.448 | -0.038 | 0.804 | -0.122 | 0.419 | 0 | 0.998 | 0.044 | 0.77 |
| OPL | 0.017 | 0.91 | -0.026 | 0.863 | -0.229 | 0.125 | 0.08 | 0.598 | -0.193 | 0.198 |
| ONL | -0.12 | 0.427 | -0.088 | 0.562 | 0.201 | 0.179 | 0.066 | 0.663 | -0.249 | 0.095 |
| RPE | 0.005 | 0.975 | 0.052 | 0.73 | 0.257 | 0.085 | 0.123 | 0.417 | -0.078 | 0.606 |
| pRNFL-G | 0.22 | 0.142 | 0.359 | 0.014* | 0.301 | 0.042* | 0.174 | 0.247 | 0.011 | 0.941 |
| pRNFL-S | 0.255 | 0.088 | 0.244 | 0.102 | 0.277 | 0.063 | 0.093 | 0.537 | -0.028 | 0.851 |
| pRNFL-I | 0.228 | 0.127 | 0.249 | 0.095 | 0.187 | 0.212 | 0.111 | 0.464 | -0.049 | 0.748 |
| pRNFL-N | 0.13 | 0.39 | 0.315 | 0.033* | 0.12 | 0.425 | 0.02 | 0.893 | 0.026 | 0.866 |
| pRNFL-T | 0.1 | 0.51 | -0.016 | 0.914 | 0.154 | 0.308 | 0.251 | 0.093 | -0.047 | 0.756 |

MRT, mean retinal thickness; mRNFL, macular retinal nerve fiber layer; GCL, ganglion cell layer; IPL, inner plexiform layer; INL, inner nuclear layer; OPL, outer plexiform layer; ONL, outer nuclear layer; RPE, retinal pigment epithelium; pRNFL, peripapillary RNFL; G, global thickness; S, superior quadrant; I, inferior quadrant; N, nasal quadrant; T, temporal quadrant; LGN, lateral geniculate nucleus; V1, primary visual cortex; FA, fractional anisotropy; fALFF, fractional amplitude of low frequency fluctuations. * denotes *p* < 0.05.
